# Influence of women empowerment on childhood (12–23 months) immunization coverage: Recent evidence from 17 sub-Saharan African countries

**DOI:** 10.1186/s41182-023-00556-2

**Published:** 2023-11-14

**Authors:** Abigail Amoah, Jacob Issaka, Castro Ayebeng, Joshua Okyere

**Affiliations:** 1 Department of Science, Jasikan College of Education, Jasikan-Buem, Ghana; 2 Department of Population and Health, College of Humanities and Legal Studies, University of Cape Coast, Cape Coast, Ghana; 3 Department of Research and Advocacy, Challenging Heights, Winneba, Ghana; 4 School of Nursing and Midwifery, College of Health Sciences, Kwame Nkrumah University of Science and Technology, Kumasi, Ghana

**Keywords:** Women empowerment, Child health, Immunization, Cross-sectional, Public health

## Abstract

**Background:**

There is a global consensus that child immunization plays an important role in promoting the health and well-being of children. Despite the quintessential role of immunization, not all children receive full immunization coverage. We examined the association between women empowerment and childhood immunization coverage in sub-Saharan Africa (SSA).

**Methods:**

The most recent Demographic and Health Survey data of 17 SSA countries were used for the analysis, with a sample of 19,223. The outcome and exposure variables were full immunization coverage and women empowerment, respectively. Full immunization was computed from percentage of children between the ages of 12 and 23 months who had received the following vaccines at any point in time: one dose of Bacille Calmette–Guérin, three doses of the vaccine protecting against diphtheria, pertussis, and tetanus or the tetravalent/pentavalent vaccine, three doses of the polio vaccine, and one dose of the measles vaccine (either as a standalone measles vaccine or as part of a combination with other immunogens). Women’s empowerment was an index of labour participation, acceptance towards spousal violence, decision-making capacity and general knowledge level. Descriptive analysis and multilevel logistic regression were performed. Results were reported in adjusted odds ratio with a corresponding 95% confidence interval.

**Results:**

The study found that 56.6% of children were fully immunized. Children of employed mothers were 1.16 times more likely to be fully immunized. Children of mothers with higher acceptance toward violence were less likely to be fully immunized [aOR = 0.90, CI 0.81, 0.99]. The odds of full immunization were higher among children born to mothers with high [aOR = 1.11, CI 1.01, 1.22] decision-making capacity. Higher odds of full immunization were found among children born to mothers with medium [aOR = 1.24, CI 1.13, 1.36] to high [aOR = 1.44, CI 1.27, 1.63] general knowledge level.

**Conclusions:**

We conclude that empowering women through livelihood empowerment interventions can increase their decision-making capacity and foster their resolve to ensure the full immunization of their children. This can be achieved by consciously investing in initiatives such as vocational training programs, job placement services, or support for entrepreneurship initiatives to encourage and support women's workforce participation.

## Background

There is a global consensus that child immunization plays an important role in promoting the health and well-being of children. Immunization provides a cost-effective means of protecting children against vaccine-preventable diseases (VPDs) such as measles, polio, tuberculosis, tetanus, and Diphtheria [1, 2]. Not only does immunization reduce children’s risk of VPDs; it is estimated that for every $1 invested in immunization, $16 health expenditure is saved [1]. Between 2011 and 2020, 24% of under-five mortality declined due to vaccines and immunization coverage [3, 4].

Despite the quintessential role of immunization, not all children receive full immunization coverage [5]. In sub-Saharan Africa (SSA), a little over half of the population of children aged 12–23 months (59.4%) had full immunization coverage [6]. The immunization coverage in SSA is further coupled with substantial economic, gender, education and residence-related inequalities [5–7]. For instance, a study by Okyere et al. [7] revealed that despite the decline in childhood immunization over time in Ghana, coverage is significantly low among children in poorer households and among male children compared to female children.

Previous literature indicates that childhood immunization coverage is influenced by a plethora of individual, community and contextual factors. Low maternal and paternal educational status [6], far distance to the nearest healthcare facility [7], residing in rural areas [8], and having home birth delivery [9] are associated with less likelihood of having complete immunization coverage. In addition, some studies [6, 10] have found high socioeconomic status, antenatal care (ANC) and postnatal care (PNC) attendance to be positively associated with childhood immunization coverage. While these individual factors show a significant association with childhood immunization coverage, the underlying factor could be women empowerment.

Higher educational attainment, higher wealth status, ANC and PNC attendance are known factors that create an enabling environment for women to be equipped with information, improve their capacity to comprehend health messages and make informed healthcare decisions that are free from coercion [11, 12]. This means that women empowerment (i.e., measured by labour force participation, attitudes to violence, decision-making, and knowledge level) may significantly predict childhood immunization coverage. A study from Pakistan [13] supports the hypothesis that there is a significant association between women empowerment and childhood immunization coverage. However, the current literature on childhood immunization coverage in SSA has been silent on this topic. The extant literature has mainly focused on the determinants of full immunization coverage [6] and inequalities in childhood immunization coverage [7]. To the best of our knowledge, there are few studies in SSA that have examined the association between women empowerment and childhood immunization coverage. However, these studies were limited to only a few indicators of empowerment [14, 15]. For instance, Singh et al.’s [14] study was limited to autonomy and attitudes to violence, while Wado et al.’s [15] was limited to autonomy. Also, the existing studies in SSA have been conducted in individual countries including Nigeria [14], Ethiopia [15] and Kenya [16]. As such, the dynamics and nuances from a regional perspective remain understudied. This knowledge gap calls for a more comprehensive study that includes multiple empowerment indicators and a regional outlook. Therefore, we examined the association between women empowerment and childhood immunization coverage in SSA. Findings from this study have implications for policy and programs that seek to improve childhood immunization coverage in SSA.

## Methods

### Data sources

**Fig. 1:**
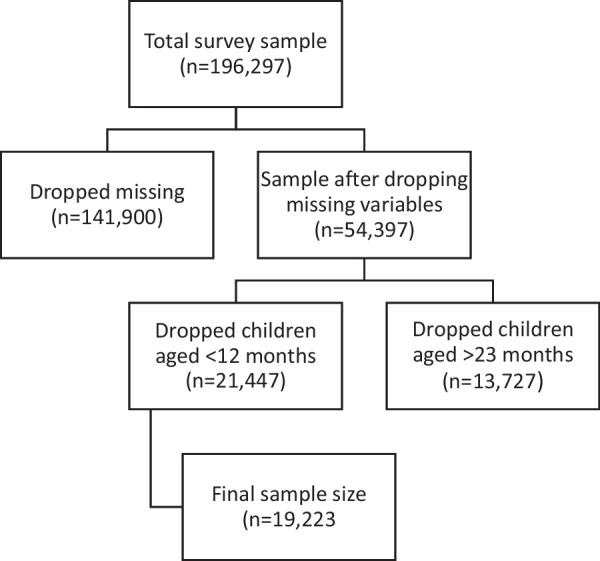
Flow chart of the sample determination

This study employed a publicly available dataset from the most recent Demographic and Health Surveys (DHS) conducted between 2017 and 2022 in sub-Saharan African countries. In reference to the defined timeframe and context, 18 countries have available DHS dataset. These countries include Burkina Faso, Benin, Cote d’Ivoire, Cameroon, Ethiopia, Gabon, Gambia, Guinea, Kenya, Madagascar, Mali, Mauritania, Nigeria, Rwanda, Sierra Leone, Senegal, and Zambia. Ethiopia was excluded from this study remaining 17 countries due to the absence of some relevant variables used in generating the main independent variable in the data. The DHS data is cross-sectional and nationally representative data from probability multi-stage samples of households in low- and middle-income countries (LMICs). Within selected households, women between the ages of 15 and 49 were asked to provide information regarding their households, personal details, and their children. The surveys use comparable methodologies for data collection and similar questionnaires that allow comparisons across countries [17, 18]. Using the child file (KR file), this study included a weighted sample of 19,223 children from the ages of 12–23 months and information on the outcome of interest (see Fig. 1). Ethical clearance was the responsibility of the institutions that administered the surveys and all analyses relied on anonymized datasets.

### Study variables and measurements

#### Outcome variable

The focus of this study was to assess full immunization coverage as the key outcome variable. Full immunization coverage, in this context, refers to the percentage of children between the ages of 12 and 23 months who have received the following vaccines at any point in time: one dose of Bacille Calmette–Guérin (BCG), three doses of the vaccine protecting against diphtheria, pertussis, and tetanus (DPT) or the tetravalent/pentavalent vaccine, three doses of the polio vaccine, and one dose of the measles vaccine (either as a standalone measles vaccine or as part of a combination with other immunogens) [19]. The outcome was dichotomised with “1” representing children who met the above definition and “0” if otherwise. In each of the countries under study, vaccines of interest were typically administered during the first year of a child's life. Data regarding the vaccines given were collected from vaccination cards, as well as from mothers’ reports for children who either lacked vaccination cards or had no recorded vaccination history [19].

#### Explanatory variables

This study’s primary factor under examination was women empowerment, as indicated by various measures. This aligns with previous research by Yaya et al. [20] and Adde et al. [21], who also considered four empowerment indicators. These indicators encompassed: (1) labour participation—this indicator classified individuals into two categories, those who were “not working = 0” and those who were “employed = 1”; (2) acceptance towards spousal violence; (3) Decision-making capacity; and (4) General knowledge level. Specifically, acceptance towards spousal violence was a composite variable derived from reasons for justification of beating a wife due to 1. wife goes out without permission; 2. wife neglects the children; 3. wife argues with husband/partner; 4. wife refuses to have sex with husband/partner; 5. wife burns the food. These were measured as yes = 1 or no = 0. An index was then created using the egen command in Stata by summing these responses, resulting in scores ranging from 0 to 5. We categorized the scores as “low” (0–1), “medium” (2–3), or “high” (4–5) acceptance of spousal violence. The Cronbach’s alpha, a measure of internal consistency, was 0.88, indicating good reliability.

Similarly, general knowledge level was a composite variable derived from 1. Level of education (no education = 0, primary = 1, secondary = 2, and higher = 3); 2. Frequency of listening to the radio (not at all = 0, at less than once a week = 1, at least once a week = 2, almost every day = 3); 3. Frequency of reading newspapers/magazines (not at all = 0, at less than once a week = 1, at least once a week = 2, almost every day = 3); 4. Frequency of watching television (not at all = 0, at less than once a week = 1, at least once a week = 2, almost every day = 3). Responses were recoded as “no” (0) or “yes” (1–3) for each factor. We created an index using the egen command in Stata by summing these responses, yielding scores ranging from 0 to 4. We categorized the scores as “low” (0), “medium” (1–2), or “high” (3–4) general knowledge level. The Cronbach’s alpha for this variable was 0.69, indicating acceptable reliability.

Also, decision-making capacity was a composite variable which gauged decision-making authority in various domains, such as who usually decides on 1. Respondent’s healthcare; 2. household earnings; 3. household purchases; 4. visits to family or relatives. These were measured as respondent alone = 0, respondent and husband/partner = 1, husband/partner alone = 2, someone else = 3, and other = 4. We recoded the responses as “yes” (0–1) or “no” (2–4) and created an index using the egen command in Stata by summing these responses, resulting in scores ranging from 0 to 3. We categorized the scores as “low” (0), “medium” (1–2), or “high” (3) decision-making capacity. The Cronbach’s alpha for this variable was 0.84, indicating good reliability. For the categorization of decision-making capacity, acceptance of spousal violence, and general knowledge level, the study followed an established division into low, medium, and high categories, as previously defined by Yaya et al. [20] and Adde et al. [21].

Multiple potential maternal, child and paternal factors informed by empirical literature relating to childhood immunization [19, 22–24] were identified and adjusted for in the regression analysis as covariates. These include age (15–19, 20–24, 25–29, 30–34, 35–39, 40–44, 45–49), type of residence (urban, rural), religion (no religion, Christianity, Islam, other religion), wealth index (poorest, poorer, middle, richer, richest), marital status (married, living with partner), age at first birth (< 20 years, 20–24, 25 and above), parity (1, 2, 3, 4 and above), number of antenatal care visits (no visit, 1–3, 4 and above), place of delivery (home, health facility), sex of child (male, female), paternal educational attainment (no education, primary, secondary, and higher), and country variable.

### Statistical analysis

We conducted a descriptive analysis of the percentage of children aged 12–23 months who were fully immunized among the studied population across the seventeen countries included in the study. A stacked bar graph was used to visually present the percentage of children who received vaccine types across the studied countries. Again, we descriptively presented the proportional distribution of vaccination type by background characteristics. A chi-square test was performed to determine statistically significant associations between the outcomes and explanatory variables. Furthermore, since the data had a hierarchical structure where children and women were nested within a cluster, we employed a more complex model (multilevel logistic regression) rather than the conventional logistic regression model. we constructed four separate multilevel logistic regression models: Model I is the null model which includes no explanatory variable, Model II included the women empowerment indicators, and Model III adjusted for other relevant maternal factors, child, and paternal factors. Lastly, the community-level factors (residence and country) were accounted for in the final model (Model IV). The models were used to calculate unadjusted (Model 1) and adjusted (Model 2–4) odds ratios with 95% confidence intervals indicating the strength of association. Log-likelihood (LL) test, Akaike's Information Criterion (AIC) and Bayesian Information Criterion (BIC) were employed to assess the fitness of various models. The model characterized by the lowest value of the information criterion will be chosen as the best model in the analysis. Prior to fitting the multilevel logistic regression model, we assessed the possibility of multicollinearity using the variance inflation factor (VIF), which revealed a mean score of 5.69, indicating the absence of significant multicollinearity. All statistical analyses were performed using Stata version 14.

### Fixed and random effect estimates

The analysis incorporated both fixed and random effects. In the fixed effect analysis, we considered a set of variables, including both individual and community-level factors. On the other hand, the random effect analysis focused on assessing the variations between clusters (EAs). To quantify these variations, we calculated two important metrics: the Intra-class correlation coefficient (ICC) and the proportional change in variance (PCV). The ICC is a measure of how much variation exists within clusters, specifically the variation between individuals within the same cluster. It was computed using the formula:

$${\text{ICC}} = {{\text{V}}_{\text{A}}}/\left( {{{\text{V}}_{\text{A}}}\, + \,{\pi ^2}/3} \right) = {{\text{V}}_{\text{A}}}/\left( {{{\text{V}}_{\text{A}}} + 3.29} \right),$$

where V_A_ is the estimated variance in each model [6].

To evaluate the total variation attributed to individual and community-level factors in each model, we used the proportional change in variance (PCV). PCV was computed as:

$${\text{PCV}} = \left( {{{\text{V}}_{\text{A}}} - {{\text{V}}_{\text{B}}}} \right)/{{\text{V}}_{\text{A}}},$$

where V_A_ is the variance of the initial model, and V_B_ is the variance of the model with additional terms [6].

## Results

### Descriptive results

Table 1 illustrates the composition of the children under study, both in terms of weighted and unweighted samples. Additionally, it provides information on the survey years, ranging from 2017/2018 to 2022. Out of the total sample of 19,223 children, 10,872 were identified as having complete immunization.

**Table 1:** Distribution of children aged 12–23 months fully immunized women in 17 SSA countries

Countries	Survey year	Unweighted (N)	Weighted (N)	Sample of children fully immunized (n)
Burkina Faso	2021	1047	1052	763
Benin	2017–2018	2073	2104	1205
Cote d’Ivoire	2021	802	749	287
Cameroon	2018	713	773	422
Gabon	2019–2021	543	615	291
Gambia	2019–2020	658	591	509
Guinea	2018	637	632	122
Kenya	2022	2777	2533	1781
Liberia	2019–2020	319	257	140
Madagascar	2021	886	899	459
Mali	2018	1622	1766	812
Mauritania	2019–2021	1286	1312	491
Nigeria	2018	2160	2240	763
Rwanda	2019–2020	618	648	623
Sierra Leone	2019	688	676	388
Senegal	2019	1008	967	737
Zambia	2018–2019	1386	1406	1076
All countries			19,223	10,872

**Fig. 2:**
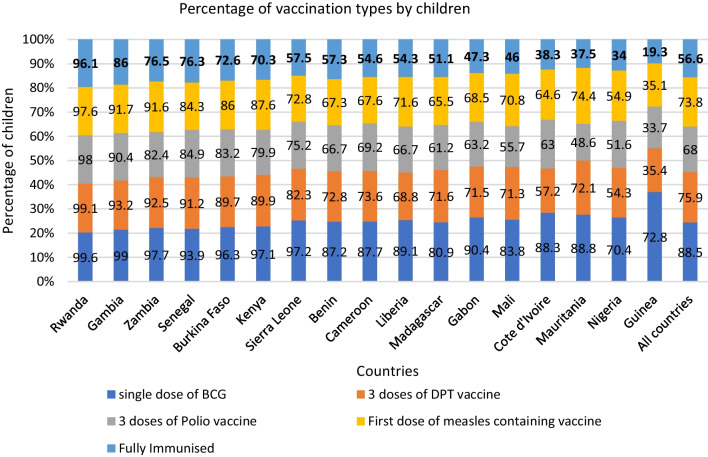
A stacked bar graph showing the percentage distribution of vaccination types among children across the 17 SSA countries

Figure 2 illustrates the percentage distribution of vaccination types among children in the 17 countries examined in this study. In total, 56.6% of the children in the study were fully immunized. The extent of full immunization varies significantly, ranging from as high as 96.1% in Rwanda to 19.3% in Guinea. Notably, Rwandan children exhibit the highest immunization rates across the four key vaccine types, with 99.6% having received a single dose of BCG, 97.6% receiving first dose of measles-containing vaccine (MCV1), 99.1% completing the 3 doses of the DPT vaccine, and 98% receiving 3 doses of the polio vaccine. Conversely, children in Guinea lag behind in nearly all vaccine categories, with only 72.8.3% having received a single dose of BCG, 35.1% receiving MCV1, 35.4% completing 3 doses of the DPT vaccine, and 33.7% receiving 3 doses of the polio vaccine.

#### Proportional distribution of vaccination type by background characteristics

Table 2 displays the findings of the analysis, indicating the proportion of fully immunised children based on various background characteristics. This includes the chi-square test score and corresponding significance levels between the explanatory variables and the outcome (full immunization). Except for the sex of the child, all women empowerment indicators together with other explanatory variables exhibited statistically significant associations with the outcome. The analysis revealed that 58.3% of children whose mothers were employed were fully immunized compared to 53.9% among those whose mothers were not employed. Regarding the perspective on violence, the majority of children (57.9%) whose mothers held a less accepting attitude towards violence were fully immunized, whereas 51.8% of those whose mothers held a more accepting attitude were fully immunized. In terms of decision-making capability, 60.8% of children whose mothers had high decision-making capacity were fully immunized, which was notably higher compared to those whose mothers had limited decision-making capacity (50.5%). A similar pattern emerged when considering the general knowledge level.

**Table 2:** Proportional distribution of vaccination type by background characteristics

Explanatory variables	Single dose of BCG [n (%)]	3 doses of DPT [n (%)]	3 doses of polio [n (%)]	First dose of measles-containing vaccine (MCV1) [n (%)]	Fully immunized [n (%)]
Women empowerment indicators					
Labour force participation	P < 0.001	P < 0.001	P < 0.001	P = 0.001	P < 0.001
Not employed	6700 (87.3)	5734 (74.7)	5030 (65.5)	5630 (73.3)	4141 (53.9)
Employed	10,312 (89.3)	8856 (76.7)	8037 (69.6)	8561 (74.1)	6731 (58.3)
Acceptance towards violence	P < 0.001	P < 0.001	P < 0.001	P < 0.001	P < 0.001
Low	11,312 (89.8)	9741 (77.3)	8717 (69.1)	9532 (75.6)	7304 (57.9)
Medium	2816 (88.8)	2415 (76.1)	2167 (68.3)	2302 (72.6)	1782 (56.2)
High	2876 (83.5)	2434 (70.7)	2182 (63.3)	2357 (68.4)	1786 (51.8)
Decision-making capacity	P < 0.001	P < 0.001	P < 0.001	P < 0.001	P < 0.001
Low	5037 (83.4)	4176 (69.2)	3781 (62.6)	4054 (67.1)	3047 (50.5)
Medium	4920 (88.9)	4204 (76.0)	3830 (69.2)	4111 (74.3)	3169 (57.3)
High	7054 (92.2)	6210 (81.2)	5456 (71.3)	6026 (78.8)	4656 (60.8)
General knowledge level	P < 0.001	P < 0.001	P < 0.001	P < 0.001	P < 0.001
Low	2561 (73.2)	2021 (57.8)	1891 (54.1)	1945 (55.6)	1397 (39.9)
Medium	9109 (89.4)	7757 (76.2)	7037 (69.1)	7536 (74.0)	5843 (57.4)
High	5342 (96.4)	4811 (86.8)	4138 (74.7)	4709 (85.0)	3631 (65.5)
Covariates					
Maternal age	P < 0.001	P < 0.001	P < 0.001	P < 0.001	P < 0.001
15–19	876 (81.3)	712 (64.9)	654 (60.7)	694 (62.2)	516 (47.8)
20–24	3669 (88.7)	3190 (75.5)	2873 (69.5)	3023 (71.8)	2355 (56.9)
25–29	4829 (89.5)	4126 (76.1)	3675 (68.1)	4041 (74.0)	3100 (57.4)
30–34	3767 (89.4)	3226 (76.6)	2890 (68.6)	3166 (74.0)	2406 (57.1)
35–39	2642 (88.7)	2277 (76.4)	2020 (67.8)	2245 (73.7)	1710 (57.4)
40–44	1000 (87.6)	880 (77.1)	790 (69.2)	839 (73.8)	649 (56.8)
45–49	228 (82.5)	179 (65.0)	163 (59.1)	181 (65.5)	135 (49.1)
Type of residence	P < 0.001	P < 0.001	P < 0.001	P < 0.001	P < 0.001
Urban	6537 (93.7)	5588 (80.1)	4858 (69.6)	5649 (78.4)	4101 (58.8)
Rural	10,475 (85.5)	9002 (73.5)	8208 (67.0)	8722 (71.2)	6771 (55.3)
Religion	P < 0.001	P < 0.001	P < 0.001	P < 0.001	P < 0.001
No religion^a^	394 (73.4)	334 (62.4)	295 (55.1)	309 (57.6)	238 (44.4)
Christians	8396 (94.5)	7535 (84.8)	6804 (76.6)	7237 (81.5)	5902 (66.4)
Islam	6700 (82.9)	5475 (67.7)	5072 (62.8)	5375 (66.5)	4017 (49.7)
Other religion^b^	379 (87.0)	320 (73.5)	277 (63.5)	312 (71.7)	239 (54.9)
Wealth status	P < 0.001	P < 0.001	P < 0.001	P < 0.001	P < 0.001
Poorest	3417 (79.0)	2849 (65.8)	2693 (62.2)	2741 (63.0)	2086 (48.2)
Poorer	3447 (84.8)	2950 (72.6)	2692 (66.2)	2854 (69.6)	2194 (54.0)
Middle	3464 (90.8)	2979 (78.1)	2655 (69.6)	2876 (74.8)	2237 (58.6)
Richer	3415 (93.9)	2939 (80.8)	2570 (70.7)	2844 (77.0)	2189 (60.2)
Richest	3268 (96.7)	2873 (85.0)	2457 (72.7)	2876 (86.4)	2165 (64.1)
Marital status	P < 0.001	P = 0.176	P < 0.001	P = 0.878	P = 0.015
Married	14,538 (88.0)	12,479 (75.6)	11,106 (67.3)	12,173 (73.7)	9243 (56.0)
Living with partner	2474 (91.4)	2111 (78.0)	1961 (72.9)	2018 (74.5)	1.629 (60.2)
Age at first birth	P < 0.001	P < 0.001	P < 0.001	P < 0.001	P < 0.001
Below 20 years	9183 (86.0)	7727 (72.4)	6980 (65.4)	7479 (70.1)	5633 (52.8)
20–24	5784 (91.5)	5077 (80.4)	4509 (71.4)	4922 (77.9)	3865 (61.2)
25 and above	2045 (91.7)	1785 (80.0)	1578 (70.7)	1789 (80.2)	1374 (61.6)
Parity	P < 0.001	P < 0.001	P < 0.001	P < 0.001	P < 0.001
1	3235 (91.3)	2866 (80.9)	2551 (72.0)	2781 (78.5)	2191 (61.8)
2	3435 (90.1)	2957 (77.5)	2670 (70.0)	2894 (75.9)	2251 (59.0)
3	2981 (89.7)	2546 (76.6)	2266 (68.2)	2518 (75.8)	1911 (57.5)
4 and above	7360 (86.2)	6220 (72.8)	5579 (65.3)	5997 (70.2)	4518 (52.9)
ANC visits	P < 0.001	P < 0.001	P < 0.001	P < 0.001	P < 0.001
No visit	748 (45.0)	546 (32.9)	579 (34.9)	555 (33.4)	346 (20.8)
1–3	5176 (89.2)	4321 (74.4)	3880 (66.8)	4222 (72.7)	3185 (54.9)
4 and above	11,088 (94.3)	9723 (82.7)	8607 (73.2)	9414 (80.1)	7341 (62.4)
Place of delivery	P < 0.001	P < 0.001	P < 0.001	P < 0.001	P < 0.001
Home	3211 (68.3)	2484 (52.8)	2383 (50.6)	2427 (51.6)	1667 (35.4)
Health facility	13,801 (95.1)	12,106 (83.4)	10,684 (73.6)	11,764 (81.0)	9205 (63.4)
Child factors					
Sex of child	P = 0.538	P = 0.542	P = 0.505	P = 0.100	P = 0.612
Male	8775 (88.5)	7521 (75.8)	6737 (67.9)	7371 (74.3)	5617 (56.6)
Female	8236 (88.5)	7069 (76.0)	6329 (68.0)	6820 (73.3)	5255 (56.5)
Paternal factors					
Educational level	P < 0.001	P < 0.001	P < 0.001	P < 0.001	P < 0.001
No education	6416 (81.2)	5240 (66.3)	4732 (59.9)	5103 (64.6)	3737 (47.3)
Primary	3942 (91.4)	3467 (80.4)	3189 (73.9)	3346 (77.6)	2713 (62.9)
Secondary	4755 (94.3)	4192 (83.2)	3655 (72.5)	4055 (80.4)	3108 (61.7)
Higher	1900 (96.5)	1690 (85.9)	1490 (75.7)	1687 (85.7)	1313 (66.7)

P: p-value

No religion^a^ (Nigeria, Zambia and Gambia do not have information on this category in the dataset)

Other religion^b^ (Sierra Leone does not have information on this category in the dataset)

Regarding the covariates, the proportions of children fully immunized significantly varied across different age groups. The lowest proportion of fully immunized children was observed among mothers aged 15–19 (47.8%), while high proportions were observed among children whose mothers were aged 20–24 to 40–44. Notably, immunization coverage was low among children whose mothers had no religious affiliation, with the highest proportion found among those whose mothers were Christians (66.4%). The proportions of children with full immunization increase with an increasing wealth index of their mothers. Compared to married women (56.0%), children whose mothers were living with a partner in cohabiting relations had a higher proportion of immunization. Higher proportions of children whose fathers had higher levels of education (66.7%) were fully immunized compared to those whose fathers had no formal education (47.3%). Most children who were delivered at the health facility (63.4%) were fully immunized than those who were delivered at home (35.4%).

#### Association between women empowerment and childhood immunization coverage

Table 3 presents the findings of the multilevel logistic regression analysis investigating the link between women empowerment indicators and childhood immunization coverage. In the full model, after adjusting for other maternal and paternal factors, child factors and community-level factors (residence and country variable), the analysis shows that children whose mothers were employed were 1.16 times more likely to be fully immunized than those whose mothers were not employed. Conversely, children whose mothers exhibited higher acceptance toward violence [aOR = 0.90, CI 0.82, 0.99] were less likely to be fully immunized than those with less acceptance toward violence. Additionally, children benefited from their mothers’ high [aOR = 1.11, CI 1.01, 1.22] decision-making capacity, and medium [aOR = 1.24, CI 1.13, 1.36] to high [aOR = 1.44, CI 1.27, 1.63] levels of general knowledge, as they had higher odds of being immunized compared to their counterparts with limited decision-making capacity and low general knowledge levels, respectively.

**Table 3:** Multilevel logistic regression analysis on association between women empowerment and childhood immunization coverage in SSA countries

Explanatory variables	Full immunization			
	Model I (null model)	Model II	Model III	Model IV (full model)
		Unadjusted OR	Adjusted OR (aOR)	Adjusted OR (aOR)
Women empowerment indicators				
Labour force participation				
Not employed		1.00	1.00	1.00
Employed		1.18*** [1.11,1.25]	1.02 [0.95,1.09]	1.16*** [1.08,1.25]
Acceptance toward violence				
Low		1.00	1.00	1.00
Medium		1.03 [0.95,1.12]	1.07 [0.98,1.17]	1.01 [0.92,1.11]
High		0.96 [0.89,1.04]	1.04 [0.96,1.14]	0.90* [0.81,0.99]
Decision-making capacity				
Low		1.00	1.00	1.00
Medium		1.21*** [1.12,1.31]	1.06 [0.97,1.16]	1.08 [0.99,1.19]
High		1.26*** [1.18,1.36]	1.06 [0.98,1.16]	1.11* [1.01,1.22]
General knowledge level				
Low		1.00	1.00	1.00
Medium		1.89*** [1.75,2.04]	1.42*** [1.30,1.55]	1.24*** [1.13,1.36]
High		2.76*** [2.52,3.02]	1.66*** [1.47,1.86]	1.44*** [1.27,1.63]
Maternal age				
15–19			1.00	1.00
20–24			1.32** [1.12,1.54]	1.27** [1.08,1.50]
25–29			1.50*** [1.25,1.80]	1.45*** [1.20,1.76]
30–34			1.60*** [1.30,1.97]	1.47*** [1.18,1.82]
35–39			1.70*** [1.36,2.12]	1.60*** [1.27,2.02]
40–44			1.75*** [1.37,2.25]	1.52** [1.17,1.97]
45–49			1.44* [1.02,2.03]	1.29 [0.90,1.85]
Religion				
No religion^a^			1.00	1.00
Christians			1.42*** [1.17,1.72]	1.20 [0.98,1.47]
Islam			0.95 [0.78,1.15]	0.82 [0.67,1.02]
Other religion			1.07 [0.82,1.40]	0.88 [0.66,1.16]
Wealth status				
Poorest			1.00	1.00
Poorer			1.05 [0.95,1.15]	1.18** [1.07,1.31]
Middle			1.07 [0.97,1.18]	1.28*** [1.15,1.42]
Richer			0.96 [0.86,1.07]	1.25** [1.11,1.42]
Richest			1.08 [1.94,1.23]	1.52*** [1.31,1.77]
Marital status				
Married			1.00	1.00
Living with partner			0.81*** [0.73,0.88]	0.94 [0.84,1.04]
Age at first birth				
Below 20 years			1.00	1.00
20–24			1.15** [1.06,1.24]	1.10* [1.01,1.20]
25 and above			0.95 [0.83,1.09]	0.95 [0.82,1.09]
Parity				
1			1.00	1.00
2			0.76*** [0.68,0.86]	0.78*** [0.69,0.88]
3			0.77*** [0.67,0.88]	0.80** [0.70,1.92]
4 and above			0.69*** [0.59,0.80]	0.73*** [0.63,0.86]
ANC visits				
No visit			0.27*** [0.23,0.31]	0.30*** [0.26,0.35]
1–3			0.87*** [0.81,0.93]	0.82*** [0.76,0.89]
4 and above			1.00	1.00
Place of delivery				
Home			1.00	1.00
Health facility			2.15*** [1.99,2.35]	1.52*** [1.39,1.67]
Sex of child				
Male			1.00	1.00
Female			0.98 [0.92,1.04]	0.98 [0.92,1.05]
Father’s education				
No education			1.00	1.00
Primary			1.13* [1.03,1.24]	1.14* [1.03,1.26]
Secondary			0.89* [0.80,0.98]	1.17** [1.05,1.30]
Higher			0.96 [0.83,1.11]	1.39*** [1.19,1.62]
Community-level factors				
Type of residence				
Urban				1.00
Rural				1.13* [1.03,1.23]
Country variable				
Burkina Faso				0.15*** [0.10,0.24]
Benin				0.08*** [0.05,0.12]
Cote d’Ivoire				0.04*** [0.02,0.06]
Cameroon				0.07*** [0.04,0.10]
Gabon				0.03*** [0.02,0.05]
Gambia				0.37*** [0.23,0.59]
Guinea				0.02*** [0.01,0.03]
Kenya				0.09*** [0.06,0.13]
Liberia				0.05*** [0.03,0.09]
Madagascar				0.06*** [0.03,0.09]
Mali				0.07*** [0.04,0.10]
Mauritania				0.02*** [0.02,0.04]
Nigeria				0.03*** [0.02,0.04]
Rwanda				1.00
Sierra Leone				0.08*** [0.05,0.13]
Senegal				0.24*** [0.15,0.37]
Zambia				0.14*** [0.09,0.21]
Random effect results				
Variance (SE)	0.107 (0.016)*	0.095 (0.015)*	0.103 (0.018)*	0.083 (0.017)*
ICC (%)	3.16	2.82	3.04	2.47
PCV (%)	1.00	11.21	− 8.42	19.42
Model fit statistics				
Log-likelihood	− 13,151.862	− 12,815.663	− 11,130.718	− 10,288.565
AIC	26,307.72	25,649.33	22,331.44	20,679.13
BIC	26,323.45	25,720.1	22,604.25	21,076.66

*1.00* reference category, *SE* Standard error, *ICC* Intraclass correlation, *PCV* Proportional change in variance, *AIC* Akaike Information Criterion; Bayesian Information Criterion

^***^p < 0.001, **p < 0.010, *p < 0.050

Turning to the variables that were controlled for, maternal age, type of residence, wealth index, age at first birth, parity, ANC visits, place of delivery, and paternal education were significantly associated with childhood immunization coverage. Consequently, when compared to children born to mothers aged 15–19, those aged 20–24, 25–29, 30–34, 35–39 and 40–44 years were 27%, 45%, 47%, 60% and 52% more likely, respectively, to be fully immunized. The analysis revealed that the likelihood of a child being fully immunized increases with an improvement in the household’s wealth status. Thus, children from households with the highest wealth index were 52% more likely to be fully immunized compared to those from the poorest households. Similarly, compared to children born to mothers who gave birth before the age of 20, those whose mothers gave their first birth between the ages of 20–24 were more likely to be fully immunized [aOR = 1.10, CI 1.01, 1.20]. Notably, children born to mothers with high parity (4 or more children) had lower odds of being fully immunized [aOR = 0.73, CI 0.63, 0.86] compared to those with uniparous mothers. The findings also highlighted a significant positive link between maternal ANC visits and place of delivery and the outcome. Hence, children whose mothers had 1–3 [aOR = 0.82, CI 0.76, 0.89] and no ANC visits [aOR = 0.30, CI 0.26, 0.35] were less likely to be fully immunized compared to their counterparts whose mothers had four or more ANC visits. We also found that children who were delivered at the health facility had 52% higher likelihood of being fully immunized compared to those who were delivered at home. Likewise, we observed increasing odds of a child being fully immunized with an increasing paternal educational level with 39% higher likelihood among those whose father had higher education than their counterparts whose father had no formal education. Furthermore, the analysis also demonstrated that children in rural areas had higher odds of being fully immunized [aOR = 1.13, CI 1.03, 1.23] in contrast to their urban counterparts. Lastly, in reference to Rwanda, children from all the countries studied were less likely to be fully immunized.

#### Random effects results

The finding revealed a significant variation in full childhood immunization across the clusters. The intra-class correlation coefficients of the null model showed that ~ 3.2% of the variation in full childhood immunization was related to community-level factors. Upon introducing both individual and community-level factors into the analysis, a statistically significant variation in full childhood immunization rates is observed across communities or clusters. The Proportional Change in Variance (PCV) for this model is approximately 19.4%, signifying that roughly 19.4% of the cluster variance initially observed in the null model can be accounted for by a combination of community and individual-level variables (see Table 3).

#### Model fit statistics

The model fitness statistics assess the overall fit of the multilevel logistic regression model. We used the Log-likelihood (LL) test, Akaike's Information Criterion (AIC) and Bayesian Information Criterion (BIC) for model comparison. Lower AIC and BIC values indicate a better fit. Remarkably, in this study, Model 4 has the lowest AIC score (20,679.13) and BIC (21,076.66), signifying that it fits the data better than the other models.

## Discussion

This study examined the association between women empowerment and childhood immunization coverage in SSA. Our study found a moderately low proportion of childhood immunization coverage across the seventeen SSA countries. The observed proportion of 56.6% aligns with the findings of Fenta et al. [6] that a little over half of children aged 12–23 months were fully immunized in SSA. This is lower than the immunization coverage of South-East Asian countries (85%) [25].

Evidence from our study supports the hypothesis that women empowerment is significantly associated with childhood immunization coverage. This is consistent with previous studies from Nigeria [14], Ethiopia [15] and Kenya [16] that have found a significant association between women empowerment indicators and childhood immunization coverage. Our study revealed that children born to mothers who were currently employed were 1.16 times more likely to be fully immunized compared to children whose mothers were unemployed. The observed association is incongruent when compared to a study from Ghana that found no significant association [26]; nonetheless, it is analogous to a study conducted in Nigeria [27] that found a positive association between employment status and childhood immunization coverage. A plausible explanation for this observation is that mothers who are employed might have higher household incomes and improved socioeconomic status. This could lead to better access to healthcare facilities, as they may have the financial means to afford the cost of transportation and other ancillary health expenditures compared to children whose mothers are unemployed. Another perspective is that mothers who are employed might have their own income, which grants them financial autonomy [28]. This financial autonomy can directly influence their decision to seek healthcare services for their children, including immunizations. Such mothers can act promptly without waiting for their partners’ approval or the need to gather funds, which can sometimes lead to delays in seeking healthcare.

We found a significant positive association between decision-making capacity and childhood immunization coverage. Children whose mothers scored medium to high decision-making capacity were more likely to be fully immunized. This aligns with Wado et al. [15] who found childhood immunization coverage to be high when mothers were able to make healthcare decisions. Similar observations have been reported by Seidu et al. [29] whose study revealed that children whose mothers had high decision-making scores were 1.3 times more likely to be fully immunized. Women with high decision-making power tend to have the freedom to decide how to use household resources, hence increasing their autonomy. This autonomy enjoyed by women serves as a motivating factor that informs them to seek healthcare for their children, including having full immunization. Merten et al. [30] argue in most societies, including SSA, that the family describes vaccination as a ‘feminised’ task. As such, mothers are tasked with the responsibility of making decisions relating to the child’s immunization. Therefore, where the woman is able to exercise this decision-making power, the more likely their children would have full immunization coverage.

The third indicator of empowerment, general knowledge level, showed a significant positive association with childhood immunization. Children whose mothers scored high knowledge levels were more likely to have full immunization. Our finding is corroborated by previous studies conducted in SSA [6] and Ghana [8]. Having a high knowledge of childhood immunization implies that mothers would have a better appreciation of the relevance of vaccination, the vaccination schedule and the possible adverse health effects that are likely to occur in the event of incomplete immunization. Previous studies [31, 32] have shown that there are normative cultural beliefs and misperceptions that serve as a barrier to childhood immunization. However, mothers who have high knowledge are informed and would be less likely to comply with the existing cultural norms that hinder childhood immunization.

Our study shows an inverse association between acceptance toward violence and full immunization coverage. That is, women who had favourable or supportive attitudes to violence were less likely to have full immunization coverage for their children. Our result is similar to Singh et al.’s [14] study that found the odds of full childhood immunization to be 1.47 times higher among children whose mothers never endorsed wife beating compared to those who found wife beating to be acceptable. This finding is accepted because women who have a supportive attitude towards violence may feel threatened to make decisions regarding the healthcare of their children out of fear that they might be abused by their partners.

Beyond the main hypothesis, we found some significant associations across the covariates. Notably, the odds of full immunization were positively associated with age—a result that corroborates previous literature [5, 6]. We also found higher odds of full immunization coverage among children in rural areas compared to those in urban areas. This is inconsistent with studies conducted in SSA [6] and Ethiopia [33]. Rural communities often have close-knit social structures were information spreads through word-of-mouth and community engagement. In such environments, peer influence and communal support might lead to higher compliance with immunization schedules. It is also possible that in rural areas, healthcare providers might have more personalized interactions with families due to smaller patient loads. This provides an easy avenue to educate and raise awareness about the need to have full immunization.

Consistent with previous literature [6, 8, 10], we found a higher likelihood of full immunization among children born to mothers in higher wealth index compared to those in the poorest wealth index. Accessing child immunization services comes with some indirect or ancillary healthcare costs such as transportation costs [34, 35]. Such costs are often cited as reasons for incomplete childhood immunization. However, being of the highest wealth offers the mother the financial resources and capacity to afford the cost of transportation and other related costs.

Our study also shows that higher frequency of ANC attendance and delivery in a healthcare facility was associated with higher odds of getting full immunization for the child. This finding agrees with studies from Bangladesh [5] and SSA [6]. Seeking maternal healthcare services (i.e., ANC and institutional birth delivery) offers an opportunity for healthcare providers to educate the woman about their responsibility to ensure full immunization of the child. Relatedly, partner educational attainment was positively associated with full immunization. The observation is expected as highly educated partners would value the significance of full immunization and support their partners in taking the child to receive full immunization. However, we found that multiparous women had lower odds of getting full immunization for their children compared to uniparous women. It is possible that multiparous women may perceive themselves as being experienced with childbirth and childrearing and, hence, may downplay the essence of child immunization.

### Strengths and limitations

In this study, we used a large nationally representative dataset. This gives us the statistical power to extrapolate our findings to the larger population of children aged 12–23 months in SSA. Arguably, this study is the first in SSA to comprehensively investigate the association between women empowerment and childhood immunization coverage from a regional perspective. This makes significant contributions to the current scholarship on childhood immunization. However, the cross-sectional nature of the data used precludes us from making any causal inferences. At best, we are able to only infer associations. We were limited to variables in the dataset, hence, other important covariates such as cultural norms and belief systems could not be accounted for.

## Conclusion

We conclude that women empowerment is positively associated with childhood immunization coverage in SSA. Recognizing this association, the study calls for multi-faceted interventions that address employment opportunities, decision-making capacity, knowledge enhancement, attitudes towards violence, and community engagement. Therefore, empowering women through livelihood empowerment interventions can increase their decision-making capacity and foster their resolve to ensure the full immunization of their children. Strengthening the knowledge level of women is needed to make them more assertive in availing their children for full immunization. This can be achieved by consciously investing in initiatives such as vocational training programs, job placement services, or support for entrepreneurship initiatives to encourage and support women’s workforce participation. Moreover, community sensitization could be implemented to promote joint decision-making between partners and empower women to take more active roles in healthcare choices, including immunization. Encouraging women to attend ANC and delivery in a healthcare facility would go a long way to improve full immunization for children in SSA. It is critical for all actions and programs to prioritize high-risk sub-populations including adolescent mothers, multiparous women, children born into poor households, and those residing in urban areas.

## Data Availability

The dataset(s) supporting the conclusions of this article is(are) available in the DHS repository at: http://dhsprogram.com/data/available-datasets.cfm

## Abbreviations

aOR: Adjusted Odds Ratios
AIC: Akaike Information Criterion
ANC: Antenatal care
BCG: Bacille Calmette–Guérin
DPT: Diphtheria, pertussis, and tetanus
DHS: Demographic and Health Surveys
SSA: Sub-Saharan African
VIF: Variance inflation factor
